# Single-image phase retrieval using an edge illumination X-ray phase-contrast imaging setup

**DOI:** 10.1107/S1600577515008978

**Published:** 2015-06-25

**Authors:** Paul C. Diemoz, Fabio A. Vittoria, Charlotte K. Hagen, Marco Endrizzi, Paola Coan, Emmanuel Brun, Ulrich H. Wagner, Christoph Rau, Ian K. Robinson, Alberto Bravin, Alessandro Olivo

**Affiliations:** a Department of Medical Physics and Biomedical Engineering, University College London, London WC1 E6BT, UK; b Research Complex at Harwell, Oxford Harwell Campus, Didcot OX11 0FA, UK; c Institute for Clinical Radiology, Ludwig-Maximilians-University, Munich 81377, Germany; d Department of Physics, Ludwig-Maximilians-University, Garching 85748, Germany; e European Synchrotron Radiation Facility, Grenoble 38043, France; f Diamond Light Source, Harwell Oxford Campus, Didcot OX11 0DE, UK; g London Centre for Nanotechnology, London WC1 H0AH, UK

**Keywords:** X-ray imaging, phase-contrast imaging, phase retrieval, image formation theory

## Abstract

A method is proposed which enables the retrieval of the thickness or of the projected electron density of a sample from a single input image acquired with an edge illumination phase-contrast imaging setup. The method assumes the case of a quasi-homogeneous sample, *i.e.* a sample with a constant ratio between the real and imaginary parts of its complex refractive index. Compared with current methods based on combining two edge illumination images acquired in different configurations of the setup, this new approach presents advantages in terms of simplicity of acquisition procedure and shorter data collection time, which are very important especially for applications such as computed tomography and dynamical imaging. Furthermore, the fact that phase information is directly extracted, instead of its derivative, can enable a simpler image interpretation and be beneficial for subsequent processing such as segmentation. The method is first theoretically derived and its conditions of applicability defined. Quantitative accuracy in the case of homogeneous objects as well as enhanced image quality for the imaging of complex biological samples are demonstrated through experiments at two synchrotron radiation facilities. The large range of applicability, the robustness against noise and the need for only one input image suggest a high potential for investigations in various research subjects.

## Abstract 2

A method enabling the retrieval of thickness or projected electron density of a sample from a single input image is derived theoretically and successfully demonstrated on experimental data.

## 1. Introduction   

X-ray imaging is an essential tool for sample inspection in several fields, including industrial testing, materials science, small-animal imaging and clinical diagnostics. In this context, X-ray phase-contrast imaging (XPCi) has demonstrated an ability to provide improved contrast for materials made of low atomic number elements, such as biological soft tissues, where attenuation differences can be limited (Bravin *et al.*, 2013 ▸; Wilkins *et al.*, 2014 ▸; Snigirev *et al.*, 1995 ▸; Davis *et al.*, 1995 ▸; Olivo *et al.*, 2001 ▸; Pfeiffer *et al.*, 2006 ▸). Among the various XPCi techniques developed so far, edge illumination (EI) has shown significant promise both in synchrotron and laboratory implementations (Olivo *et al.*, 2001 ▸; Olivo & Speller, 2007 ▸; Munro *et al.*, 2012 ▸; Diemoz, Endrizzi *et al.*, 2013 ▸; Diemoz, Hagen *et al.*, 2013 ▸; Munro *et al.*, 2013 ▸; Hagen *et al.*, 2014 ▸), due to the simplicity and flexibility of the experimental setup and its practically negligible requirements in terms of spatial and temporal coherence (Olivo & Speller, 2007 ▸; Munro *et al.*, 2012 ▸; Diemoz, Hagen *et al.*, 2013 ▸). However, these practical advantages do not come at the expense of the phase sensitivity provided by EI, which was shown to be comparable with or even better than other XPCi techniques (Diemoz, Endrizzi *et al.*, 2013 ▸; Diemoz, Hagen *et al.*, 2013 ▸).

Like other XPCi approaches, such as analyzer-based imaging (ABI) (Davis *et al.*, 1995 ▸; Chapman *et al.*, 1997 ▸) and grating interferometry (GI) (Pfeiffer *et al.*, 2006 ▸), the images acquired with an EI setup contain a mixture of attenuation and refraction (or differential phase) contrast, the latter being proportional to the spatial derivative of the X-ray phase shift. Methods that enable the separation and evaluation of these two quantities have been developed (Munro *et al.*, 2012 ▸; Diemoz, Endrizzi *et al.*, 2013 ▸; Diemoz, Hagen *et al.*, 2013 ▸; Munro *et al.*, 2013 ▸) which, however, require two images acquired in different configurations of the setup as input for the retrieval algorithm. While retrieval methods making use of a single experimental image have been proposed for other XPCi techniques (Paganin *et al.*, 2002 ▸, 2004 ▸; Burvall *et al.*, 2011 ▸; Nesterets *et al.*, 2004 ▸; Pavlov *et al.*, 2004 ▸; Briedis *et al.*, 2005 ▸; Momose, 2002 ▸; Momose *et al.*, 2009 ▸), based on a variety of different assumptions and implementations, a single-image retrieval method for EI has not been developed yet. Such a method would be preferable in order to reduce the duration of the acquisition, a key requirement in many applications such as computed tomography (CT). Moreover, the existing implementations of EI do not provide the phase map directly, but rather its first derivative, which often has a significant intensity only along the boundaries of the sample details. Retrieval of the phase map would be advantageous in cases where subsequent processing (*e.g.* segmentation) is required, or where the object structure is complex (as is typical for many biological samples), in order to enable an easier image interpretation. While in principle the phase map could be obtained through one-dimensional integration of the refraction image (Hasnah *et al.*, 2005 ▸), this procedure is known to produce strong streak artefacts along the integration direction, due to propagation of the noise in the refraction image. This is a well known problem of differential XPCi techniques, and various algorithms have been developed to try to reduce this effect, both in ABI and GI XPCi (Wernick *et al.*, 2006 ▸; Thüring *et al.*, 2011 ▸).

In this article, we propose a method that enables direct retrieval of the phase map from a single EI image. The method is shown to produce artefact-free images, and to combine quantitative accuracy and robustness to noise.

## 2. Theory   

**Figure 1:**
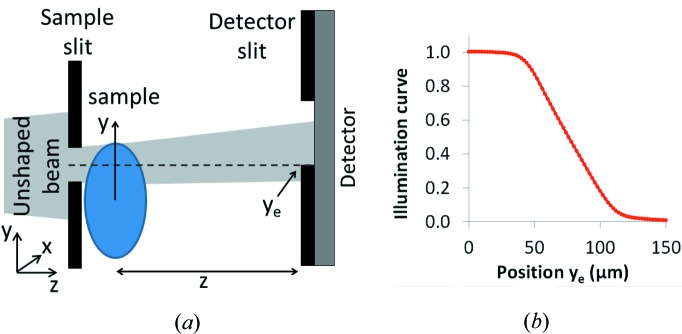
(*a*) Scheme of the EI experimental setup (diagram not to scale). (*b*) Example of illumination curve, measured at the ESRF ID17 beamline at an energy of 27 keV.

The EI working principle is schematically presented in Fig. 1 ▸(*a*). The incoming beam is collimated in one direction by a first slit (with apertures typically from a few to a few tens of microns) located before the sample. A second slit, placed in front of the detector, is partially misaligned with respect to the first: as a result, part of the beam is stopped by the slit, while the remaining fraction impinges on the detector. The X-ray refraction introduced by the object leads to a spatial shift of the beam position at the detector plane, the component of which along the direction *y* orthogonal to the slits is equal to 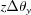, where *z* is the object-to-detector distance and 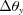
is the refraction angle along *y*. This beam shift will cause either an increase or a decrease of the photons counted by the detector, depending on the direction of refraction [see Fig. 1 ▸(*a*)].

In order to obtain a full image of the sample, a scanning of the latter along *y* needs to be performed. This scanning procedure can be avoided, in the case of a large beam covering the whole object (*e.g.* from a conventional X-ray tube), by replacing the slits with masks that replicate the EI principle over the entire field of view (Olivo & Speller, 2007 ▸).

If the object refraction angle and transmission are approximately constant within the height of the first aperture, the signal recorded by the detector along *y* is equal to (Diemoz, Endrizzi *et al.*, 2013 ▸; Diemoz, Hagen *et al.*, 2013 ▸)
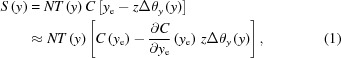
where *N* is the total number of photons passing through the first aperture and *y* represents the sampling position in the object. 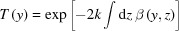 is the transmission and 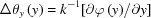 is the refraction angle, where 
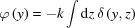
 is the phase shift, *k* is the X-ray wavenumber and 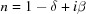 is the complex refractive index. The illumination curve 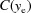 represents the fraction of the unperturbed beam entering the detector aperture, as a function of the position 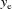 of the latter, and is obtained by scanning one of the slits vertically. An example of an illumination curve [measured at the European Synchrotron Radiation Facility (ESRF), see below for details on the experimental setup] is reported in Fig. 1 ▸(*b*). The right-hand side of equation (1) is obtained through a first-order Taylor expansion, in the approximation that the beam shift due to refraction is small compared with the width of the illumination curve (Munro *et al.*, 2013 ▸).

In the direction parallel to the slits, however, the recorded signal is the same as that obtainable in free-space propagation (FSP) (Diemoz *et al.*, 2014 ▸). If the object attenuation and phase are varying sufficiently slowly and the propagation distance is not too long (near-field regime) (Gureyev *et al.*, 2008 ▸), this can be expressed by the transport-of-intensity equation (Teague, 1983 ▸). By combining the expressions for the signal in both directions, the normalized signal 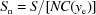 can be written as
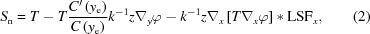
where, for simplicity of notation, we have dropped the dependence of *S*_n_, *T* and 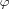 upon the object coordinates *x* and *y*; 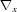 and 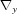 indicate derivation with respect to *x* and *y*, and 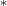 indicates convolution. LSF_*x*_ is the line spread function of the imaging system along the *x* direction, which takes into account the blurring due to both the projected source size and the detector point spread function (Gureyev *et al.*, 2008 ▸). In EI, instead, it can be shown that the effect of the source blurring on the signal is already taken into account by the shape of the illumination curve (Diemoz, Hagen *et al.*, 2013 ▸), while the detector point spread function does not affect the signal (Diemoz *et al.*, 2014 ▸). It can be seen from equation (2) that the signal depends on the two (unknown) functions *T* and 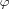, which are in turn dependent on the distributions of β and δ. The number of unknown quantities, however, reduces to one if the ratio 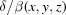 can be considered constant across the object. Although this simplifying assumption is strictly valid only in the case of a sample made of a single material, extensive use of it has been made in the literature (Paganin *et al.*, 2002 ▸; Pavlov *et al.*, 2004 ▸; Briedis *et al.*, 2005 ▸). This approximation was shown, in fact, to provide good results in several practical cases (Paganin *et al.*, 2002 ▸; Pavlov *et al.*, 2004 ▸; Briedis *et al.*, 2005 ▸; Sanchez *et al.*, 2012 ▸), and to be well suited in particular for soft biological tissues, which feature very similar chemical compositions (Olendrowitz *et al.*, 2012 ▸; Wernersson *et al.*, 2013 ▸). For simplicity, we will first consider the special case of a homogeneous sample with constant values for β and δ, as used by Paganin *et al.* (2002 ▸). Under this assumption, 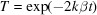 and 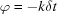, where the object thickness function *t* is now the unknown quantity to be determined. The following results will be then generalized in the case of the more relaxed assumption of constant 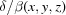.

We follow here an approach analogous to those employed by Paganin *et al.* (2002 ▸), Pavlov *et al.* (2004 ▸) and Briedis *et al.* (2005 ▸) for the FSP and ABI XPCi techniques. If we introduce the definition 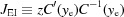, equation (2) can be rewritten as
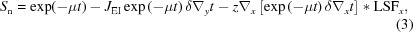
where 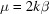 is the linear attenuation coefficient. By noting that 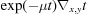 = 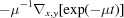 and by developing the second and third terms accordingly, equation (3) can be rewritten in a more compact form:
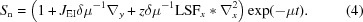
We now take the two-dimensional Fourier transform of both sides of equation (4) and make use of the Fourier derivative theorem, which gives:
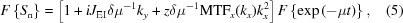
where *F* indicates the two-dimensional Fourier transform, 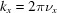 and 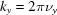, where 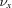 and 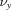 are the Fourier space coordinates, and 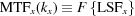 is the system modulation transfer function along the *x* direction. A single input image *S*_n_ allows solving the above equation for the unknown quantity *t*,
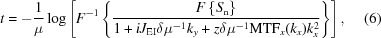
where 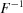 indicates the inverse Fourier transform. Equation (6) can be implemented efficiently by means of the fast Fourier transform. A similar expression for the projected electron density 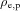 can be obtained under the more relaxed assumption of constant δ/β ratio. In fact, by noting that the line integral of δ is equal to 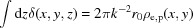, where 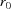 is the classical electron radius (Born & Wolf, 1980 ▸), and following an approach analogous to that used in equations (3)–(6), it is found that
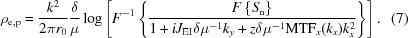



## 3. Experimental results   

We now present two experimental demonstrations of the method, obtained with different setups, to highlight the method’s flexibility and wide range of applicability. The first experiment was carried out at the ID17 beamline of the ESRF (Grenoble, France). The source size is about 132 µm (horizontal) × 24 µm (vertical) (full width at half-maximum), and is located approximately 140 m from the experimental hutch. An energy of 27 keV was selected by using a double-crystal Si(111) monochromator in Laue geometry. The tungsten slits are oriented horizontally at a mutual distance of 8.90 m; their apertures are 20 µm and 250 µm, respectively. An illumination level of 50% [*i.e.* *C*(*y*_e_) = 0.5] was used for the acquisitions, corresponding to the lower edge of the second slit being aligned with the centre of the first slit [see Fig. 1 ▸(*a*)]. A custom-made phantom consisting of wires of known materials is used to demonstrate the method’s quantitative accuracy for a single-material object. The sample is placed on a motorized translation stage 3.85 m upstream of the second slit, and scanned vertically with steps of 20 µm during the acquisition. The images are acquired with a FReLoN CCD camera (Coan *et al.*, 2006 ▸), with an effective pixel size of 46 µm × 46 µm and 1 s exposure time.

**Figure 2:**
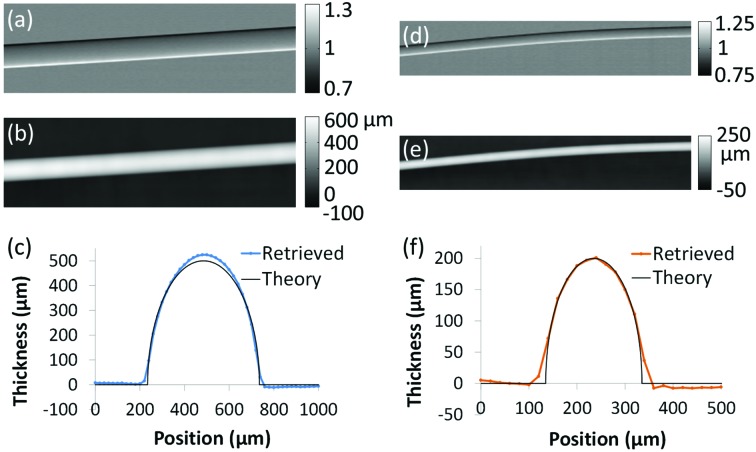
(*a*) Mixed EI image, (*b*) retrieved thickness and (*c*) corresponding vertical profile for the 500 µm PET wire. (*d*) Mixed EI image, (*e*) retrieved thickness and (*f*) vertical profile of thickness for the 200 µm PEEK wire.

The ‘raw’ images containing a mixture of attenuation and refraction contrast are shown in Figs. 2 ▸(*a*) and 2(*d*). The first wire is made of polyethylene terephthalate (PET) and has a diameter of 500 µm; the second is made of polyether ether ketone (PEEK) and has a diameter of 200 µm. It can be noted that the amplitude of the FSP signal is significantly smaller than that of the EI signal, due primarily to the relatively large pixel size, which blurs the FSP signal [*cf*. equation (2)]. For each of the two images, equation (6) was used to retrieve the object thickness map. The following nominal values were considered in the calculation: δ = 4.09 × 10^−7^ and β = 7.83 × 10^−11^ for PET, δ = 3.92 × 10^−7^ and β = 6.91 × 10^−11^ for PEEK (Dejus & Sanchez del Rio, 1996 ▸). The retrieved thickness maps for the PET and PEEK wires are shown in Figs. 2(*b*) and 2(*e*) ▸, respectively, and the corresponding vertical profiles in Figs. 2(*c*) and 2(*f*) ▸. The expected thickness profiles are also shown for comparison: they assume perfectly cylindrical wires with a diameter equal to the nominal one provided by the supplier.

There is reasonable agreement between retrieved and nominal thickness for both wires, and good image quality is obtained for the retrieved images in Figs. 2 ▸(*b*) and 2(*e*). In particular, the vertical streak artefacts visible when the phase map is obtained from integration of the refraction image are suppressed. This can be mainly attributed to the additional filtering along *x* present in equation (6): although in this experimental layout the FSP signal along *x* is limited, the filter effectively enforces consistency between columns, thus greatly reducing the vertical streak artefacts.

The second experiment demonstrates the applicability of the method under very different experimental conditions, and its benefits for the imaging of more complex biological samples. It was performed at beamline I13 (coherence branch) of the Diamond Light Source (Didcot, UK) using an X-ray energy of 9.7 keV. This energy is selected through a horizontally deflecting Si(111) pseudochannel-cut crystal monochromator. The source full width at half-maximum is equal to about 400 µm (horizontal) × 13 µm (vertical); the experimental hutch is located about 220 m from the source. The first slit, made of gold electroplated on a silicon substrate, is oriented horizontally and has an aperture equal to 3 µm. In this experiment, the method described by Vittoria *et al.* (2014 ▸) was used, where the second slit is replaced by a high-resolution detector. This is a PCO Edge camera, consisting of a scintillator, magnifying visible light optics and an sCMOS sensor: it was operated with an 8× magnification, which provides an effective pixel size of 0.8 µm. A ‘virtual’ edge is created through multiplication of the acquired frame by a Heaviside function, chopping the illuminated area in half along the vertical direction (Vittoria *et al.*, 2014 ▸). The distance between sample slit and sample was equal to 5 cm, while the sample-to-detector distance was 30 cm. The sample is a flower petal with superimposed pollen grains. The vertical scan step was 1.6 µm, and the exposure time 7 s.

**Figure 3:**
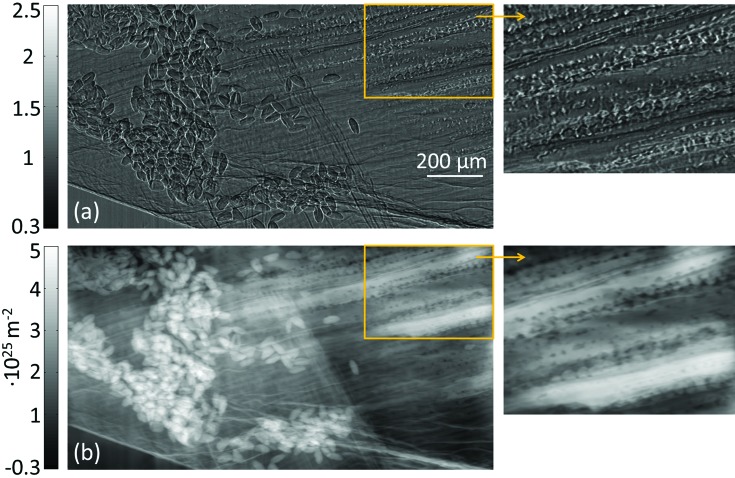
EI images of a flower petal and pollen grains: (*a*) mixed image, (*b*) retrieved map of the projected electron density.

In this case, the exact sample materials are unknown, and the retrieval of the projected electron density 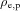 was thus performed by using δ/β as a tunable parameter. A δ/β ratio corresponding to that of water was first assumed (δ = 2.46 × 10^−6^ and β = 5.94 × 10^−9^) (Dejus & Sanchez del Rio, 1996 ▸), then adjusted to obtain the best observable image quality (achieved with a δ/β ratio equal to about 0.8 times that of water). The mixed image and extracted map for 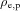 are presented in Figs. 3 ▸(*a*) and 3(*b*). It can be seen that, owing to the very small pixel size employed, the amplitudes of the FSP and EI signals in the mixed image (respectively along the horizontal and vertical directions) are comparable in this case. Indeed, under conditions of very high coherence and very small pixel size the advantages of EI over FSP tend to be reduced. The pollen grains are clearly visible in the left region of the images. Cells lining up along the veins of the petal can also be seen. They show up as dark spots in the 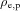 image, because their density is lower than that of the surrounding tissue [see enlarged region of Fig. 3 ▸(*b*)]. Although in this case the sample materials do not strictly satisfy the assumption of constant δ/β ratio, meaning that the estimated 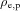 values should be interpreted with caution, the obtained map is free from image artefacts and useful for interpreting the complex structure of the sample. In particular, it provides complementary information to the mixed one. While the latter is superior in terms of visualization of the smaller structures, corresponding to higher frequencies, low object spatial frequencies are better highlighted in the former. This can also prove useful for subsequent processing, such as segmentation.

**Figure 4:**
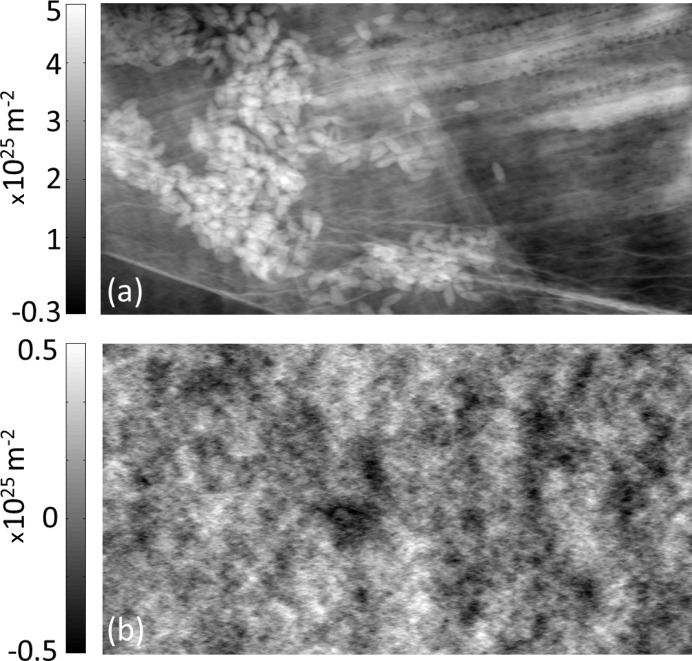
(*a*) Retrieved phase map of the flower sample, obtained after adding 30% Poisson noise to the image in Fig. 3(*a*). (*b*) Difference between phase maps in Figs. 3(*b*) and 4(*a*) (note the different color scale used).

Finally, a test of the method’s robustness with respect to noise was carried out. Poisson noise corresponding to statistics of only 10 photons per pixel (standard deviation ∼30%) was added numerically to the image of the petal before the retrieval. Despite the very high noise in the input image, the retrieved map of 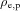 still maintains its ability to correctly visualize most of the sample structures, as seen in Fig. 4 ▸(*a*). The difference between the retrieved maps obtained with low (Fig. 3 ▸ *b*) and high (Fig. 4 ▸ *a*) levels of noise is presented in Fig. 4 ▸(*b*) (note that a different color scale has been used since the values are small).

The high stability with respect to noise, apparent from Figs. 4 ▸(*a*) and 4(*b*), can be explained by the fact that equations (6) and (7) behave as low-pass filters, thus largely suppressing high-frequency noise. At the same time, however, low-frequency artefacts are also limited since the filter never diverges (in particular at the zeroth frequency), leading to all spatial frequencies being well behaved. It is worth noting that this property is a direct result of exploiting both attenuation and refraction information from the input image (the attenuation signal effectively acts as a regularization term, by imposing point-wise consistency between absorption and retrieved 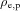 values).

## 4. Conclusions   

The method proposed in this article has been shown to provide retrieved images of high quality, to be robust against noise and free from the streak artefacts often encountered with differential phase methods. Only one input image is required, which is advantageous in terms of reduced exposure time and radiation dose to the sample. If the ratio δ/β is approximately constant and its value is known, fairly accurate quantitative information can be extracted, *i.e.* the projected electron density and, if δ and β are constant, the object thickness. Our test on a biological object shows also that the assumption of a constant δ/β does not have to be rigidly satisfied for high-quality images to be obtained. Another significant advantage over integration of differential phase images is that the method can be used on objects larger than the field of view, as prior knowledge of the phase values at the image boundaries is not required.

The developed method can be applied to both planar and CT imaging over a wide range of experimental conditions. Future work will be dedicated to extend its use to EI laboratory setups employing polychromatic beams from conventional X-ray tubes.
